# A Longitudinal Study of Sick Building Syndrome (SBS) among Pupils in Relation to SO_2_, NO_2_, O_3_ and PM_10_ in Schools in China

**DOI:** 10.1371/journal.pone.0112933

**Published:** 2014-11-14

**Authors:** Xin Zhang, Fan Li, Li Zhang, Zhuohui Zhao, Dan Norback

**Affiliations:** 1 Institute of Environmental Science, Shanxi University, Taiyuan, China; 2 School of Public Health, Fudan University, Shanghai, China; 3 Department of Medical Sciences, Occupational and Environmental Medicine, Uppsala University, Uppsala, Sweden; Peking University, China

## Abstract

There are fewer longitudinal studies from China on symptoms as described for the sick building syndrome (SBS). Here, we performed a two-year prospective study and investigated associations between environmental parameters such as room temperature, relative air humidity (RH), carbon dioxide (CO_2_), nitrogen dioxide (NO_2_), sulphur dioxide (SO_2_), ozone (O_3_), particulate matter (PM_10_), and health outcomes including prevalence, incidence and remission of SBS symptoms in junior high schools in Taiyuan, China. Totally 2134 pupils participated at baseline, and 1325 stayed in the same classrooms during the study period (2010–2012). The prevalence of mucosal symptoms, general symptoms and symptoms improved when away from school (school-related symptoms) was 22.7%, 20.4% and 39.2%, respectively, at baseline, and the prevalence increased during follow-up (*P*<0.001). At baseline, both indoor and outdoor SO_2_ were found positively associated with prevalence of school-related symptoms. Indoor O_3_ was shown to be positively associated with prevalence of skin symptoms. At follow-up, indoor PM_10_ was found to be positively associated with new onset of skin, mucosal and general symptoms. CO_2_ and RH were positively associated with new onset of mucosal, general and school-related symptoms. Outdoor SO_2_ was positively associated with new onset of skin symptoms, while outdoor NO_2_ was positively associated with new onset of skin, general and mucosal symptoms. Outdoor PM_10_ was found to be positively associated with new onset of skin, general and mucosal symptoms as well as school-related symptoms. In conclusion, symptoms as described for SBS were commonly found in school children in Taiyuan City, China, and increased during the two-year follow-up period. Environmental pollution, including PM_10_, SO_2_ and NO_2_, could increase the prevalence and incidence of SBS and decrease the remission rate. Moreover, parental asthma and allergy (heredity) and pollen or pet allergy (atopy) can be risk factors for SBS.

## Introduction

Non-specific symptoms associated with the indoor climate problems, often called sick building syndrome (SBS), have been reported in Western countries since the 1970s. The symptoms include headaches, fatigue, and irritation in the upper airways nose, throat, eyes, hands and/or facial skin [1], [2]. The symptoms can be very common in particular buildings with indoor problems and the severity may vary from person to person, even within the same building [3]. Some studies restrict SBS to symptoms improved or disappearing when away from a particular indoor environment (transient symptoms) [4] while others include all symptoms, irrespectively if reported to be improved or not [5]. Researchers from North Europe have used the later definition for long time [6].

A number of personal, organisational and environmental factors can be associated with SBS. Indoor environmental factors associated with SBS include building dampness [7]–[9], low ventilation flow [10], volatile organic compounds (VOC) [11], mold and bacteria [12], microbial VOC (MVOC) [12] and room temperature [13]–[15]. Moreover, personal factors such as female gender, history of allergic disorders and perception of odor have been demonstrated to be associated with SBS [5], [16], [17].

Since children and adolescents are more vulnerable, the school environment has been suggested to be an important indoor environment in a public health perspective [18]. One study reported a high prevalence (25.2%) of SBS among primary school students in Japan [4], and another study reported 50% of SBS in one school in Sweden [19]. Most school environment studies have been performed in North America and Europe [20]–[22]. In Hong Kong, a number of environmental exposures were measured in five classrooms. The average respirable particulate matter concentrations were higher than the HK objective, and the maximum indoor PM_10_ level exceeded 1000 µg/m^3^. Indoor CO_2_ concentrations often exceeded 1000 ppm, indicating inadequate ventilation. It was concluded that the two most important classroom air quality problems were PM_10_ and CO_2_ levels [23]. Another epidemiological study in schools in Malaysia, measuring fungal DNA, reported associations between certain microbial species (Aspergillus versicolor and Streptomyces sp) and respiratory symptoms in school children [24]. Moreover, associations between building dampness and mould in the classroom and SBS in school children have been reported in Japanese and Danish schools [4], [25].

China has the largest population in the world but there are fewer studies on risk factors for SBS symptoms in China. We have previously performed a two-year follow-up study from 2004–2006 on SBS among junior high school students in schools in Taiyuan, a coal-burning city in Northern China [26]. The study demonstrated that NO_2_ and SO_2_ levels in the classrooms were positively associated with the prevalence of SBS symptoms. Moreover, parental asthma and allergy (heredity) and pollen or pet allergy (atopy) were risk factors for both prevalence and incidence of SBS [26]. In order to further study associations between SBS and indoor and outdoor air pollution in schools, including particulate matter (PM_10_), we have performed a new two-year longitudinal school environment study six years later in the same schools. Since the first study was performed the air pollution situation in Taiyuan city has been improved.

The aim was to investigate associations between measured indoor and outdoor environment and prevalence, new onset (incidence) and remission of SBS symptoms among junior high school students in schools in Taiyuan city, Shanxi province, China. The indoor climate measurements included room temperature, relative air humidity (RH) and concentration of carbon dioxide (CO_2_). The measured indoor and outdoor air pollutants included nitrogen dioxide (NO_2_), sulphur dioxide (SO_2_), ozone (O_3_) and particulate matter (PM_10_). Finally, we studied associations between gender, parental asthma or allergy (heredity), and pollen or pet allergen (atopy) and prevalence, incidence and remission of SBS symptoms. The study included symptoms improved when away from the school (school-related symptom) as well as total prevalence, incidence and remission of symptoms.

## Materials and Methods

The study is a two-year follow-up of a cohort with repeated questionnaires in a random sample of Chinese pupils, at baseline and at follow-up, with environmental measurements at baseline.

### Study design

Ten junior high schools were randomly selected in the urban area of Taiyuan, a city with three million inhabitants. Taiyuan is the capital of Shanxi province and is located 500 km south west of Beijing. Taiyuan is one of the most heavily polluted cities in China, and Shanxi province is a major coal mining area in China. All headmasters of the selected schools agreed to participate. Data on SBS symptoms were collected by questionnaires distributed to each student in randomly selected classes by the head teacher. The study was performed in March 2010 (baseline) and was repeated two years later at follow-up (March 2012). Measured air pollutants included nitrogen dioxide (NO_2_), sulphur dioxide (SO_2_), ozone (O_3_), particulate matter (PM_10_) and measured climate factors included temperature, RH and carbon dioxide (CO_2_). Environmental measurements were performed in each selected classroom and outside each school at baseline, only, during a two-week period in March 2010. The measurements were performed just after the questionnaire study was completed. The measurement period was in the end of the heating season in Taiyuan.

### Study population

At baseline (2010), five first-year classes were randomly selected in each of the ten schools. If there were less than five first-year classes, all were selected. The study population consisted of 2335 pupils (11–15 years of age) in 44 classes, of which 2134 (91.4%) completed the questionnaire. There were no reports on health complaints or environmental problems from any of the schools before the investigation. At follow-up (2012), two schools that had been selected at baseline did not participate. In the end, 33 classes in eight schools completed the questionnaire. A total of 1325 pupils participated in both the initial study and follow-up. Air pollution and climate factors were measured in 32 classrooms with 1283 participants. Measurements could not be done in one classroom (with 42 participants) due to practical reason (lack of an electric outlet). All participating students stayed in the same classroom during lessons, except for sport, because each class had a fixed classroom during all three years of junior high school.

### Ethics statement

There was informed consent from the pupils and the parents before the study. We gave the parents, the children, and their head teacher an information letter together with the questionnaire, stating that if they answered and returned the questionnaire it meant they had given informed consent. The nature and possible consequences of the study were explained before the study began. The questionnaire study and the exposure measurements in the schools had permission from the principal of each school and the head teacher of each class involved in the study. All personal information from questionnaire was kept confidential. The study protocol and the consent procedure were approved by the Institute of High School Student Health Care in Taiyuan which did not require a written consent since the study only included questionnaires and no clinical tests.

### Questionnaire data

Students were given a self-administered questionnaire to collect data about age, sex, parental asthma or allergy (heredity), allergy, asthma, and medical symptoms compatible with SBS. Questions on allergy and respiratory health included ‘yes/no’ questions of doctor diagnosed asthma, current asthma and allergies to dog, cat or pollen. The questionnaire survey was performed one week before the classroom inspections and measurements. The questionnaires were distributed by the class teachers. The same questionnaire was used in 2010 and 2012. The questionnaire was based on previous school studies [8], [26]. SBS-symptoms included facial and hand rash or itching; eczema; eye irritation; swollen eyelids; nasal catarrh and obstruction; dryness in the throat; sore throat; irritating cough; headache; nausea; sensation of getting a cold and tiredness. The recall period was 3 months. Each question had four alternative answers: ‘Yes, everyday’; ‘Yes, 1–4 times/week’; ‘Yes, 1–3 times/month’; and ‘No, never’. In addition, a question concerning whether any of the SBS symptoms improved when they stayed away from school (school- related symptoms) was also included. In the statistical calculations, for mucosal (eye irritation, swollen eyelids, nasal catarrh, nasal obstruction, dryness in throat, sore throat or irritating cough), general (headache, nausea, sensation of getting a cold or tiredness) and dermal symptoms (facial and hand rash or itching, eczema), weekly symptom (yes everyday or days 1–4 times/week) was coded 1 and 1–3 times/month or never was coded 0. For symptoms improved when away from school, any symptoms improved were coded 1 and no symptoms improved when away from school was coded 0.

### Building inspection and climate measurements

Details on building construction, materials, pot plants, number of students and type of ventilation system were noted, as were any sign of building dampness, such as damp spots, water leakage or indoor mould growth in the classrooms. Temperature (°C), relative air humidity (RH%) and CO_2_ (ppm) concentration were measured during normal activities in the classrooms using a Q-track IAQ monitor (TSI Incorporated, St. Paul, MN, USA). Corresponding Q-track measurements were done simultaneously outside each school.

### Air pollution measurements

Indoor levels of SO_2_, NO_2_ and O_3_ were measured in the selected classrooms (N = 32) and outdoor levels were measured at one representative location in each school by diffusion samplers. The sampling time was a continuous seven-day period (24 h/day) for each sampler. Indoor samplers were placed approximately 2 m above the floor. Outdoor samplers were placed 2.5–3.5 m above the ground, under a well-ventilated plastic cover protecting them from rain and snowfall. The samplers were obtained from IVL Swedish Environmental Research Institute Ltd. (Gothenburg, Sweden). The samplers were analyzed by an accredited laboratory (IVL) specializing in analyzing such samplers, and were reported as average values across the 7-day measurement period. The concentration of indoor and outdoor PM_10_ was measured in parallel by direct DUST Track II Aerosol monitor (TSI Incorporated, St. Paul, MN, USA) during one day in each school. Temperature, relative humidity (RH), CO_2_ and PM_10_ were measured for two hours in each classroom, with a full class and during lectures. The outdoor levels were measured for one hour simultaneously the same day in each school.

### Data from local monitoring stations

In order to compare our measurement data with the general air pollution in the city, and to study time trends in air pollution, air pollution data was collected from all nine local monitoring stations in Taiyuan. Annual mean values were available for SO_2_, NO_2_ and PM_10_ for 2010, 2011 and 2012. PM_2.5_ was not measured in Taiyuan until 2013. To get information on the ratio between PM_2.5_ and PM_10_ in the city, we collected annual means for PM_2.5_ and PM_10_ for 2013. In addition, we collected monitoring station data on SO_2_, NO_2_ and PM_10_ for the study period (two weeks in March 2010).

### Data analysis

Questionnaire data was entered independently by two persons using Epidate 3.1 software. Differences in prevalence between 2010 and 2012 were tested by the McNemar test. For each student, the occurrence of any mucosal, skin, or general symptoms (weekly symptoms) was calculated in both the beginning and the end of the follow-up period. New onset (incidence) of any mucosal symptom was defined as the presence of at least one mucosal symptom at the end of the follow-up period but the absence of any mucosal symptom in the beginning. New onset of any skin or any general symptom was defined in a similar way. The reference group for calculating new onset was subjects not reporting the particular group of symptom neither in 2010 nor in 2012. Remission of a particular group of symptoms (mucosal, skin or general) was defined as presence of any symptom (e.g., mucosal) in the group in the beginning and absence of any mucosal symptom at the end of the follow-up. The reference group for calculating remission was subjects reporting the particular group of symptom in 2010 as well as in 2012. New onset and remission of SBS symptoms was defined in a similar way as in our previous study [26].

Multiple logistic regression was used to analyze associations between the dependent variables (SBS symptoms) and exposure (indoor exposure on classroom level or outdoor exposure on school level), controlling for age, sex, parental asthma or allergy, and keeping each exposure separate in the models. For all statistical analyses, a two-tailed tests and 5% significance level were applied. All statistical analyses were conducted using SPSS 20.0.

## Results

In total, 2134 of 2335 pupils (91.4%) participated in the baseline questionnaire study in 10 schools (baseline). The mean age was 13.7 years (range 11–15 years). The follow-up study was restricted to those 1325 pupils (62.1%) who participated in both the initial study and the follow-up study and who stayed in the same classroom. We found no significant differences between participants (N = 1325) and non-participants (N = 809) with respect to asthma, atopy, smoking habits,parental asthma or allergy, or prevalence of any general symptom, any mucosal symptom, any skin symptom or any symptom improved when away from school (Table 1). The mean age was 13.4 years (SD = 0.70) among participants and 14.2 years (SD = 0.96) among non-participants (*p* = 0.69). However, the proportion of girls among the participants were higher (*p* = 0.02).

**Table 1:** Demographic data from baseline study (2010) comparing questionnaire data for participants (N = 1325) and non-participants (N = 809) in the two-year follow-up study.

Symptoms	Prevalence (%)in participants(N = 1325)	Prevalence (%)in non-participants(N = 809)	*P*-value^a^
Gender (girl)	53.0	46.7	0.02
Parental allergy or asthma	9.4	8.4	0.42
Ever had asthma	0.8	1.6	0.10
Cat allergy	1.1	1.5	0.38
Dog allergy	0.6	0.5	0.74
Pollen allergy	3.3	3.1	0.77
Any skin symptoms^b^	4.1	5.4	0.18
Any mucosal symptoms^c^	21.7	24.5	0.15
Any general symptoms^d^	18.6	23.4	0.49
Any symptoms improved if away from school^e^	37.7	41.5	0.11

a Differences tested by *Chi*-square test.

b The prevalence of subjects with at least one weekly symptom classified as skin.

c The prevalence of subjects with at least one weekly symptom classified as mucosal.

d The prevalence of subjects with at least one weekly symptom classified as general.

e The prevalence of subjects with at least one of the above mentioned symptoms classified as improved if away from school.

The cumulative incidence of asthma (ever had asthma) and tobacco smoking had increased during the follow-up period and there was a significant increase of any mucosal symptom, any general symptom, any dermal symptom and any symptom that improved when away from school (school-related symptoms). Among individual symptoms, most mucosal and general symptoms had increased (Table 2). Data on new onset (incidence) and remission of symptoms is given in Table 3. The incidence during the two-year period was highest for general symptom (33%) and lowest for skin symptoms (6%). The incidence of school-related general and mucosal symptoms was 11% and 15%, respectively while the incidence of school-related skin symptoms was low (3%). The remission of symptoms during the two-year period ranged from 21–38%, with the lowest remission for general symptoms and the high remission for skin symptoms.

**Table 2:** The prevalence of asthma, allergy, smoking and weekly symptoms in the last 3 months 2010 and 2012 among junior high school students in Taiyuan.

	Participants in 2010	Follow-up study among participants in 2010 and 2012		
Symptoms	2010(N = 2134) (%)	2010(N = 1325) (%)	2012(N = 1325) (%)	*P*-value^a^
**Ever had asthma**	0.9	0.7	1.5	0.01
**Doctor’s diagnosed asthma**	1.1	0.8	1.0	0.77
**Current smoker**	0.3	0.2	0.9	0.01
**Cat allergy**	1.2	1.0	2.1	0.05
**Dog allergy**	0.6	0.6	0.8	0.61
**Pollen allergy**	3.2	3.1	4.2	0.07
**Any skin symptoms** b	4.6	3.9	6.0	0.02
Rash on hands	1.0	0.9	1.2	0.56
Facial rashes	0.7	0.7	1.4	0.11
Eczema	0.6	0.5	0.9	0.24
Facial itching	3.0	2.6	3.8	0.10
Itching on the hands	1.8	1.7	3.0	0.04
**Any mucosal symptoms** c	22.7	21.4	29.7	<0.001
Eye irritation	2.7	3.2	4.2	0.19
Swollen eyelids	1.5	1.4	2.8	0.01
Nasal catarrh	11.3	10.3	14.6	<0.001
Nasal obstruction	10.8	10.1	15.4	<0.001
Throat dryness	10.7	9.6	14.2	<0.001
Irritative cough	5.0	4.9	9.0	<0.001
**Any general symptoms** d	20.4	18.7	35.6	<0.001
Headache	4.7	3.6	8.5	<0.001
Nausea	4.3	3.5	6.6	<0.001
Sensation of getting a cold	5.1	4.8	9.5	<0.001
Tiredness	16.4	15.3	28.5	<0.001
**Symptoms improved if away from schoo1 (school-related symptoms)** e	39.2	16.2	24.1	<0.001
**Any skin school related symptoms**	2.3	0.8	2.6	0.60
**Any mucosal school related symptoms**	12.9	6.9	12.0	<0.001
**Any general school related symptoms**	11.7	6.9	15.8	<0.001

a Differences between 2010 and 2012 tested by McNemar test.

b The prevalence of subjects with at least one weekly symptom classified as skin.

c The prevalence of subjects with at least one weekly symptom classified as mucosal.

d The prevalence of subjects with at least one weekly symptom classified as general.

e The prevalence of subjects with at least one of the above mentioned symptoms classified as improved if away from school.

**Table 3:** The number of cases and percentage of new onset and remission of SBS symptoms during the two-year follow-up (N = 1325).

	Onset			Remission		
Symptoms	N^a^	N^b^	%	N^c^	N^d^	%
Any skin symptoms	75	1273	5.9	48	127	37.8
Any mucosal symptoms	267	1042	25.6	156	550	28.4
Any general symptoms	350	1077	32.5	126	598	21.1
Symptoms improved if away from school	230	1111	20.7	125	444	28.2
Any skin school related symptoms	35	1315	2.66	10	45	22.22
Any mucosal school related symptoms	136	1233	11.03	69	251	27.49
Any general school related symptoms	180	1233	14.60	63	301	20.93

a Number of new onset cases for the particular type of symptoms.

b Number of persons who did not report the particular type of symptoms at baseline or at follow-up.

c Number of remission cases for the particular type of symptoms.

d Number of persons who had the particular type of symptoms at both times.

The buildings were constructed of concrete or bricks. No classroom had any mechanical ventilation or air-conditioning, opening windows was the only means of ventilation. The windows were mostly closed because of the cold climate. The classrooms had concrete floors without any paint. The floors were cleaned one to three times per day by means of wet mopping by the pupils. No visible sign of building dampness, water leakage, mould growth or water damage was observed in any of the selected classrooms, and very few classrooms had any indoor pot plants. The average indoor concentrations of SO_2_, NO_2_, O_3_ and PM_10_ were 68.0, 43.2, 8.6 and 129 µg/m^3^, respectively (Table 4). The average indoor/outdoor ratio was 37% for SO_2_, 91% for NO_2_, 48% for O_3_ and 77% for PM_10_. When comparing exposure data at baseline for schools participating (N = 8) and not participating (N = 2) in the longitudinal study, no major differences were observed (Table 4). As a next step, correlations between indoor environmental exposures were investigated. At higher indoor relative air humidity, levels of indoor NO_2_, PM_10_ and CO_2_ were higher. At higher indoor CO_2_, levels, indoor PM_10_ were higher. Room temperature was not significantly correlated with any exposure variable. There was a close correlation between indoor NO_2_ and SO_2_, but no significant correlations between other chemical air pollutants (Table 5).

**Table 4:** Average indoor and outdoor climate and concentration of air pollutants, measured at baseline (2010).

	Total material			Classes in schoolsparticipating in 2012			Classes in schoolsnot-participating in 2012		
Type of exposures	N^a^	Mean ± SD	Min-Max	N^a^	Mean ± SD	Min-Max	N^a^	Mean ± SD	Min-Max
**Indoor climate and air pollutants in classrooms**									
Number of students	39	52±8	34–65	32	50±8	35–65	7	47±8	39–55
SO_2_ (µg/m^3^)	39	65.6±24.5	22.0–162.1	32	68.0±24.0	22.0–122.5	7	77.6±22.3	45.7–115.8
NO_2_ (µg/m^3^)	39	42.6±7.0	22.2–53.3	32	43.2±6.6	25.5–53.3	7	46.5±5.0	37.4–51.7
O_3_ (µg/m^3^)	39	8.5±8.3	2.9–44.0	32	8.6±7.4	2.9–44.0	7	8.7±4.0	3.0–13.0
PM_10_ (µg/m^3^)	39	118±103	19–332	32	129±97	19–332	7	173±32	139–223
Temperature (°C)	39	18.8±1.6	14.0–24.5	32	18.6±1.5	16.0–23.0	7	18.3±1.0	17.0–20.0
CO_2_ (ppm)	39	1289.9±609.6	428–2728	32	1208±580	428–2728	7	994.4±603.6	429–2115
Relative airhumidity (RH) (%)	39	25±9	11–44	32	24±8	11–44	7	23±7	17–35
**Climate and air pollutants outside the schools**									
SO_2_ (µg/m^3^)	10	173.3±38.5	118.5–301.4	8	183.5±49.5	118.5–280.1	2	215.1±71.3	150.0–280.1
NO_2_ (µg/m^3^)	10	46.7±3.3	39.5–56.1	8	47.5±3.4	41.8–53.3	2	50.2±1.4	48.9–51.5
O_3_ (µg/m^3^)	10	18.0±5.1	6.0–25.0	8	18.1±4.5	6.0–25.0	2	19.0±3.3	16.0–22.0
PM_10_ (µg/m^3^)	10	188±220	24–594	8	168±179	24–594	2	186±45	144–227
Temperature (°C)	10	2.7±0.6	2.2–4.4	8	2.8±0.7	2.5–4.3	2	3.9±0.4	3.5–4.3
CO_2_ (ppm)	10	413.7±59.9	321–492	8	414±51	335–489	2	404.5±13.7	392–417
Relative airhumidity (RH) (%)	10	16±8	5–26	8	15±6	6–26	2	14±5	9–18

a Number of classrooms for indoor measurements and number of schools for outdoor measurements.

**Table 5:** Correlation between indoor exposures (Pearson correlation) (N = 39).

	SO_2_	NO_2_	O_3_	PM_10_	Temperature	CO_2_	RH
SO_2_	1						
NO_2_	0.762**	1					
O_3_	0.153	0.258	1				
PM_10_	0.207	0.313	−0.219	1			
Temperature	−0.254	−0.218	−0.156	0.061	1		
CO_2_	0.324	0.289	−0.104	0.397*	0.240	1	
RH	0.502**	0.457**	−0.182	0.525**	−0.031	0.876**	1

*P<0.05.

**P<0.01.

In Taiyuan there are nine monitoring stations for outdoor air pollution. Average data for SO_2_, NO_2_ and PM_10_ during the study period (2010–2012) was given in Table 6. There was a trend of decreased pollution levels for SO_2_ and PM_10_ and increased levels of NO_2_ during the follow-up period, but the differences were not large. Moreover, we compared mean air pollution values from the monitoring station for the two week study period in March 2010 with annual means for 2010. The monitoring data for SO_2_ during the two week study period was the same (100%) as the annual mean while the level of PM_10_ during the study period were 167% higher and the level of NO_2_ was 71% lower. In China, outdoor air pollution of fine particles (PM_2.5_) is currently a hot topic. In Taiyuan, PM_2.5_ was not measured until 2013. In 2013, the annual mean of PM_2.5_ was 77.7 µg/m^3^ at the monitoring stations measuring PM_2.5_ in Taiyuan, corresponding to 49.6% of the annual PM_10_ value for 2013 at the same stations.

**Table 6:** Annual means of outdoor exposures measured at monitoring stations.

	Average of annual means (N^a^ = 9)		
	SO_2_ (µg/m^3^)	NO_2_ (µg/m^3^)	PM_10_ (µg/m^3^)
2010	68.4	20.2	88.9
2011	61.2	22.3	82.4
2012	58.3	27.8	80.3

a Number of monitoring stations in Taiyuan.

Associations between the prevalence of SBS symptoms and measured environment factors at baseline were studied by multiple logistic regression, adjusting for age, gender, parental asthma, and keeping each exposure factor separately in the models. Both indoor and outdoor SO_2_ were positively associated with any symptom improved when away from school (school-related symptoms). Indoor O_3_ was positively associated with any skin symptoms, and outdoor NO_2_ was negatively associated with any skin symptoms (Table 7).

**Table 7:** Associations between measured environmental factors and the prevalence of weekly symptoms at baseline in 2010 (N = 2134).

Type of exposures	Any skinsymptoms(OR 95% CI)	Any mucosalSymptoms(OR 95% CI)	Any generalSymptoms(OR 95% CI)	Any symptomsimproved ifaway fromschool(OR 95% CI)	Any school relatedmucosal symptoms(OR 95% CI)	Any school relatedgeneral symptoms(OR 95% CI)
**Indoor climate and air pollutants in classrooms**						
SO_2_	1.02(0.93–1.13)	0.78(0.48–1.29)	0.83(0.49–1.40)	1.83(1.18–2.83)**	1.28(0.70–2.36)	1.30(0.69–2.44)
NO_2_	0.75(0.53–1.06)	0.84(0.71–1.01)	0.86(0.72–1.04)	1.14(0.97–1.34)	1.02(0.81–1.27)	0.93(0.74–1.17)
O_3_	1.46(1.16–1.83)***	1.02(0.88–1.19)	1.10(0.94–1.28)	1.09(0.95–1.25)	1.06(0.88–1.28)	1.03(0.85–1.25)
PM_10_	0.61(0.18–2.26)	0.95(0.84–1.08)	2.08(0.55–0.78)	1.08(0.96–1.20)	1.11(0.96–1.30)	1.15(0.98–1.34)
Temperature	1.15(0.98–1.34)	0.57(0.26–1.28)	1.03(0.45–2.36)	1.00(0.86–1.17)	0.46(0.17–1.26)	1.00(0.36–2.80)
CO_2_	0.74(0.47–1.18)	0.90(0.73–1.10)	0.97(0.78–1.20)	1.01(0.85–1.21)	0.90(0.69–1.16)	0.79(0.60–1.05)
RH	0.72(0.50–1.02)	0.92(0.79–1.07)	0.96(0.82–1.12)	1.05(0.92–1.19)	0.98(0.81–1.19)	0.94(0.77–1.15)
**Air pollutants outside the school**						
SO_2_	1.16(0.72–1.87)	1.08(0.85–1.37)	1.21(0.95–1.55)	1.25(1.02–1.55)*	1.16(0.86–1.56)	1.30(0.96–1.77)
NO_2_	0.29(0.13–0.62)**	0.87(0.61–1.24)	0.91(0.63–1.32)	1.21(0.87–1.66)	0.97(0.63–1.51)	0.89(0.56–1.40)
O_3_	0.82(0.46–1.44)	0.77(0.58–1.03)	1.04(0.77–1.41)	0.96(0.74–1.24)	0.88(0.64–1.20)	0.91(0.66–1.26)
PM_10_	0.62(0.27–1.43)	0.97(0.91–1.04)	1.92(0.87–4.24)	1.40(0.69–2.82)	1.05(0.97–1.14)	1.08(0.99–1.17)

Odds ratio (OR) with 95% confidence interval (CI) calculated by multiple logistic regression. Adjustment for age, gender and parental asthma or allergy, analysing each exposure variable separately.

*P<0.05.

**P<0.01.

***P<0.001.

As a next step, associations between new onset (incidence) of SBS symptoms and the exposures were analyzed by multiple logistic regression (Table 8). Indoor PM_10_ was positively associated with new onset of skin symptoms, mucosal symptoms, general symptoms and school-related mucosal and general symptoms. Classroom CO_2_ concentration was positively associated with new onset of mucosal symptoms, general symptoms and any symptom improved when away from school, including school-related mucosal and general symptoms. Classroom relative air humidity was positively associated with new onset of mucosal symptoms, general symptoms and any symptom improved when away from school, including school-related mucosal and general symptoms. Outdoor concentration of SO_2_ was positively associated with new onset of dermal symptoms, only. Outdoor NO_2_ was positively associated with new onset of dermal, general and mucosal symptoms. Outdoor PM_10_ levels were positively associated with new onset of dermal, general and mucosal symptoms as well as any symptom improved when away from school, including school-related mucosal and general symptoms (Table 8).

**Table 8:** Associations between measured environmental factors and onset (incidence) of weekly symptoms during the two-year follow-up from 2010 to 2012 (N = 1283).

Type ofexposures	Any skinSymptoms(OR 95% CI)	Any mucosalSymptoms(OR 95% CI)	Any generalSymptoms(OR 95% CI)	Any symptomsimproved ifaway from school(OR 95% CI)	Any school relatedmucosal symptoms(OR 95% CI)	Any school relatedgeneral symptoms(OR 95% CI)
**Indoor climate and air pollutants in classrooms**						
SO_2_	0.61(0.19–2.01)	0.61(0.30–1.23)	0.92(0.49–1.74)	0.70(0.33–1.48)	0.57(0.22–1.43)	0.65(0.29–1.47)
NO_2_	0.87(0.59–1.28)	0.97(0.76–1.23)	0.98(0.78–1.22)	0.85(0.67–1.09)	0.94(0.69–1.28)	0.87(0.66–1.14)
O_3_	0.86(0.59–1.26)	0.91(0.74–1.12)	0.91(0.75–1.10)	0.88(0.70–1.11)	0.93(0.72–1.21)	0.92(0.73–1.17)
PM_10_	1.39(1.10–1.76)**	1.34(1.14–1.57)***	1.24(1.06–1.45)**	1.01(0.84–1.22)	1.33(1.09–1.63)**	1.26(1.05–1.52)*
Temperature	1.49(0.28–7.80)	1.45(0.51–4.08)	1.12(0.82–1.23)	1.21(0.95–1.35)	1.17(0.98–1.33)	1.24(0.91–1.40)
CO_2_	1.43(0.96–2.11)	1.59(1.23–2.06)***	1.85(1.45–2.36)***	1.73(1.31–2.27)***	1.93(1.43–2.62)***	1.87(1.41–2.48)***
RH	1.33(0.93–1.76)	1.47(1.22–1.76)***	1.51(1.27–1.79)***	1.41(1.16–1.71)***	1.65(1.33–2.05)***	1.56(1.28–1.91)***
**Air pollutants outside the school**						
SO_2_	2.08(1.11–3.90)*	1.33(0.90–1.97)	1.17(0.85–1.68)	0.91(0.60–1.39)	0.93(0.55–1.58)	1.05(0.66–1.67)
NO_2_	1.16(1.07–1.25)***	2.03(1.27–3.25)**	1.64(1.06–2.54)*	0.90(0.54–1.51)	1.54(0.83–2.85)	1.14(0.65–1.99)
O_3_	0.60(0.34–1.08)	1.11(0.75–1.65)	1.14(0.79–1.64)	1.47(0.94–2.29)	1.24(0.72–2.13)	1.51(0.92–2.48)
PM_10_	1.22(1.07–1.40)**	1.25(1.14–1.37)***	1.20(1.09–1.32)***	1.18(1.06–1.31)**	1.33(1.20–1.47)***	1.29(1.17–1.43)***

Odds ratio (OR) with 95% confidence interval (CI) calculated by multiple logistic regression. Adjustment for age, gender and parental asthma or allergy, analysing each exposure variable separately.

*P<0.05.

**P<0.01.

***P<0.001.

Remission of SBS symptoms in relation to air pollution levels were analyzed in the same way by multiple logistic regression (Table 9). Indoor PM_10_ level was negatively associated with remission of any mucosal symptoms and any symptom improved if away from school, including school-related mucosal and general symptoms. Classroom CO_2_ concentration and relative air humidity were negatively associated with remission of any symptom improved when away from school. Outdoor SO_2_ was negatively associated with remission of mucosal symptoms and outdoor NO_2_ and outdoor PM_10_ were negatively associated with remission of any symptom improved when away from school.

**Table 9:** Associations between measured environmental factors and remission of weekly symptoms during the two-year follow-up from 2010 to 2012 (N = 1283).

Type of exposures	Any skin symptoms (OR 95% CI)	Any mucosal symptoms (OR 95% CI)	Any general symptoms (OR 95% CI)	Any symptoms improved if away from school (OR 95% CI)	Any school related mucosal symptoms (OR 95% CI)	Any school related general symptoms (OR 95% CI)
**Indoor climate and air pollutants in classrooms**						
SO_2_	1.02(0.99–1.04)	0.80(0.27–2.35)	1.34(0.39–4.58)	1.14(0.27–4.74)	0.96(0.77–1.20)	0.98(0.78–1.25)
NO_2_	1.02(0.93–1.12)	0.97(0.66–1.42)	0.95(0.62–1.45)	0.84(0.49–1.46)	0.91(0.39–2.12)	0.69(0.29–1.63)
O_3_	0.97(0.92–1.03)	0.97(0.74–1.29)	0.96(0.71–1.29)	1.24(0.88–1.74)	1.22(1.71–2.10)	0.86(0.53–1.41)
PM_10_	1.43(0.59–3.49)	0.71(0.54–0.93)*	0.82(0.63–1.08)	0.59(0.44–0.81)**	0.52(0.31–0.88)*	0.62(0.40–0.97)*
Temperature	0.69(0.42–1.14)	0.79(0.14–4.35)	1.00(0.82–1.22)	0.96(0.77–1.20)	0.75(0.52–1.08)	1.16(0.78–1.72)
CO_2_	0.51(0.13–2.05)	0.72(0.46–1.14)	1.02(0.62–1.66)	0.60(0.37–0.99)*	1.07(0.43–2.69)	1.26(0.49–3.22)
RH	0.92(0.35–2.40)	0.81(0.58–1.13)	1.02(0.72–1.45)	0.60(0.42–0.85)**	0.93(0.48–1.79)	0.99(0.53–1.86)
**Air pollutants outside the school**						
SO_2_	1.01(0.99–1.04)	0.38(0.20–0.74)**	0.82(0.39–1.69)	0.80(0.34–1.91)	0.89(0.78–1.05)	0.93(0.81–1.07)
NO_2_	1.05(0.87–1.29)	0.50(0.24–1.04)	0.79(0.36–1.74)	0.37(0.15–0.89)*	0.91(0.79–1.04)	0.89(0.78–1.02)
O_3_	0.88(0.76–1.03)	0.80(0.41–1.54)	0.72(0.34–1.54)	0.90(0.37–2.18)	0.65(0.16–2.62)	1.03(0.88–1.20)
PM_10_	0.81(0.34–1.95)	0.83(0.68–1.01)	0.93(0.79–1.09)	0.72(0.60–0.85)***	0.78(0.58–1.04)	0.86(0.67–1.11)

Odds ratio (OR) with 95% confidence interval (CI) calculated by multiple logistic regression. Adjustment for age, gender and parental asthma or allergy, analysing each indoor climate variable separately.

*P<0.05.

**P<0.01.

***P<0.001.

Finally, associations between gender, parental allergy or asthma (heredity), pollen or pet allergen (atopy) and SBS symptoms were analysed, both at baseline and longitudinally (Table 10). In the cross-sectional analysis, girls reported less skin symptoms, less mucosal symptoms and less school-related mucosal symptoms. Parental asthma or allergy (heredity) was positively associated with all types of symptoms. In the longitudinal analysis, girls had a lower incidence of skin symptoms. Parental asthma or allergy (heredity) was associated with a higher incidence of mucosal symptoms, general symptoms and any symptom improved when away from school, including school-related mucosal and general symptoms. Pollen or pet allergy (atopy) at baseline was associated with a higher incidence of skin symptoms, only. No associations were found between personal risk factors and remission of symptoms.

**Table 10:** Associations between gender, pollen or pet allergy, parental allergy or asthma and weekly symptoms in cross-sectional and longitudinal analysis.

	Type offactors	Any skinSymptoms(OR 95% CI)	Any mucosalSymptoms(OR 95% CI)	Any generalSymptoms(OR 95% CI)	Any symptomsimproved ifaway fromschool(OR 95% CI)	Any schoolrelated mucosalsymptoms(OR 95% CI)	Any schoolrelated generalsymptoms(OR 95% CI)
**Cross-sectional** **analysis in** **2010 (N = 2134)**	Female gender	0.63(0.42–0.95)*	0.78(0.64–0.96)*	0.97(0.79–1.21)	0.96(0.80–1.15)	0.76(0.59–0.99)*	1.05(0.81–1.37)
	Pollen or petallergy	1.89(0.79–4.56)	1.32(0.77–2.25)	1.08(0.61–1.92)	1.44(0.89–2.34)	1.37(0.77–2.41)	1.95(0.94–1.33)
	Parentalallergy/asthma	1.89(1.07–3.34)*	1.57(1.13–2.17)**	1.83(1.32–2.53)***	1.64(1.22–2.21)***	1.50(1.01–2.23)*	1.85(1.25–1.73)**
**Longitudinal** **analysis in** **the follow-up** **(N = 1325)**	Incidence ofsymptoms						
	Femalegender	0.62(0.39–1.00)*	0.86(0.64–1.14)	0.87(0.67–1.13)	0.86(0.64–1.15)	0.75(0.53–1.08)	0.99(0.72–1.36)
	Pollen or petallergy	3.29(1.48–7.28)**	1.81(0.85–3.85)	1.37(0.67–2.81)	1.11(0.48–2.57)	1.04(0.49–2.23)	0.90(0.44–1.86)
	Parentalallergy/asthma	1.86(0.84–4.12)	1.95(1.24–3.07)**	1.76(1.13–2.76)**	2.08(1.29–3.37)**	2.03(1.20–3.42)**	1.81(1.10–2.96)*
	Remission ofsymptoms						
	Femalegender	1.23(0.33–4.54)	1.40(0.85–2.29)	0.80(0.48–1.36)	0.62(0.35–1.11)	0.57(0.22–1.50)	0.54(0.22–1.35)
	Pollen or petallergy	1.95(0.16–23.88)	1.37(0.42–4.49)	1.25(0.33–4.71)	0.34(0.09–1.34)	1.41(0.19–1.99)	1.06(0.66–1.25)
	Parentalallergy/asthma	1.27(0.14–11.17)	0.52(0.21–1.26)	0.82(0.35–1.92)	0.62(0.26–1.52)	0.90(0.26–3.16)	0.52(0.17–1.58)

Odds ratio (OR) with 95% confidence interval (CI) calculated by multiple logistic regression.

*P<0.05.

**P<0.01.

***P<0.001.

## Discussion

In our study among junior high school students in Taiyuan, a heavily polluted city, the prevalence of mucosal symptoms, general symptoms and school-related symptoms was relatively high at baseline and increased during the two-year follow-up. We found associations between indoor climate and ventilation and indoor and outdoor air pollution in the schools and different types of SBS symptoms. Higher concentrations of SO_2_, NO_2_, O_3_ and PM_10_ were positively associated with higher prevalence and higher incidence of SBS and negatively associated with remission of symptoms. Parental asthma or allergy (heredity), pollen or pet allergy (atopy) and male gender were important risk factors for prevalence and incidence of SBS. The study was performed in the same schools as our previous two-year longitudinal school study in Taiyuan performed in 2004–2006 [26]. Our current study could confirm findings from the previous study with respect to chemical air pollutants despite the environmental improvements that had taken place in the city. This suggests that the air pollution levels in Taiyuan in 2010–2012 were still high enough to cause impaired health. To our knowledge, our study is one of few longitudinal studies on SBS in relation to indoor and outdoor air pollution in schools in China.

Epidemiological studies can be affected by selection bias and information bias. The schools were randomly selected within Taiyuan city and first-year classes were randomly selected within the schools. The response rate in the initial study was 92%, 62% of which participated in the follow-up study. The participation rate in the follow-up was a bit low, but was mainly due to the lack of participation of two schools and the transfer of some pupils to other schools or other classrooms during follow-up. There was no indication of selection bias when comparing classes participating and not participating in the longitudinal study, except for a somewhat higher participation rate among girls. Concerning information bias, the same questionnaire was used in the current study as in the previous study from 2004–2006 [26]. The questionnaire data was collected one week before environment measurements, and exposure levels were unknown when the students answered the questionnaires, and there were no visible signs of microbial growth in any classroom. The study was done in the same month (March) both times, during normal lecturing activities. We measured chemical air pollutants using a badge type of diffusion sampler fully based on the theory for diffusion sampling [27]. With this sampler, the theoretical sampling rate can be used to calculate the pollutant concentrations [28], and movement of air inside the sampler resulting from wind induced turbulence [29] and other artifacts are minimized.

One limitation of our study is the short sampling time, 2 hours for temperature, relative air humidity, CO_2_ and PM_10_ and one week continuous air monitoring for chemical air pollutants (NO_2_, SO_2_, O_3_). Another limitation is that we only have exposure data measured at baseline. The advantage with having exposure data at baseline is that the exposure is measured before the change of symptoms. Data from local monitoring stations in the city suggests a reduction of SO_2_ and PM_10_ and an increase of NO_2_ levels during the study period but the changes were not large. Moreover, the level of SO_2_ during the two week measurement period (March 2010) was the same as the annual mean for that year but the levels of PM_10_ was higher and the levels of NO_2_ was lower when we did our measurements as compared to the annual mean. The absolute pollution levels measured during two weeks may not be representative for the long term mean exposure, but the relative ranking of the schools (rank order) can be assumed to be more stable and similar even if the absolute exposure levels vary over time. Another limitation of our study is that it focused on particular matter and chemical air pollutants, and did not measure allergens and microbial markers. In the previous study from 2004–2006, dust was collected by vacuum cleaning [8]. Some bacterial and fungal compounds were found to be protective while others were risk factors for SBS symptoms. However, since these microbial compounds are mainly from indoor sources, they should not be major confounders in our study measuring air pollutants mainly from outdoor sources. Another limitation is that we do not have data on the air pollution at home, where the children spend most of their time. However, at this age most children live near their school and it can be assumed that the general outdoor pollution levels are similar at home and at school, but ventilation habits may differ between the school and the home. Most of the limitations of our study would most likely lead to non-differential misclassification. In conclusion, we do not believe that the results and conclusions from our study were seriously biased by selection bias or information bias.

Shanxi province is a major coal mining area with one-third of China’s domestic coal production, and Taiyuan is a heavily industrialized area relying on coal combustion. The city is surrounded by mountains and has a heavy outdoor air pollution, especially during the heating season and especially for SO_2_ and particulate matter. Moreover, satellite monitoring has demonstrated that this area sometimes has one of the highest pollution levels of NO_2_ in the world [30]. In our previous school study from the same schools in 2004 [26], the weekly mean concentration of SO_2_ was 265 µg/m^3^ indoors and 713 µg/m^3^ outdoors. In our current study from 2010, the levels had decreased to 68.0 µg/m^3^ indoors and 183.5 µg/m^3^ outdoors. This is a about 4 times lower levels, suggesting a considerable environmental improvement with respect to SO_2_ levels in Taiyuan. In the previous study from 2004, the weekly mean concentration of NO_2_ was 39.4 µg/m^3^ indoors and 52.3 µg/m^3^ outdoors [26]. In our current study from 2010, levels were similar (43.2 µg/m^3^ indoors and 47.5 µg/m^3^ indoors). Finally, the weekly mean concentration of indoor O_3_ was 8.6 µg/m^3^ in our study, similar as in the previous study (10.1 µg/m^3^) in 2004. The weekly mean concentration of outdoor O_3_ was 18.1 µg/m^3^, in our study, around 1–1.5 times the outdoor levels of O_3_ (12.4 µg/m^3^) in the previous study. Our data were one week average, so they are not directly comparable with the WHO guidelines. The WHO air quality guideline for SO_2_ is 20 µg/m^3^ (24-hour mean) and 500 µg/m^3^ for a 10-min mean value. The WHO air quality guideline for NO_2_ is 40 µg/m^3^ (annual mean) and 200 µg/m^3^ for 1-hour mean. The WHO air quality guideline for O_3_ is 100 µg/m^3^ for a 8-h mean value [31]. The local government and environmental protection agency in Shanxi province have taken a series of measures to reduce level of SO_2_ and particulate matters recent years. Our pollutions measurements in the two school studies suggest that these measures have been effective. However, the levels of NO_2_, a pollutants mainly coming from vehicle exhaust, were still high and did not change over six years.

None of the schools had a mechanical ventilation system, and window opening was the only way to ventilate the classrooms. CO_2_ is an indication of ventilation flow in a room, and is related to the number of persons in the room as well as the air exchange rate. Typically, ventilation standards require that the CO_2_ level should be below 1000 ppm [32]. In the current study from 2010, the mean CO2 level was 1208 ppm but in the previous study from 2004 the mean level was much higher (2211 ppm) [26]. In the current study from 2010, both CO_2_ and RH were associated with an increased incidence of mucosal symptoms, general symptoms and symptoms improved if away from school, and decreased remission of symptoms improved if away from school. In the previous study from 2004–2006, we found negative association between indoor levels of CO_2_ and symptoms significant only found in the cross-sectional analysis [26]. One explanation of the different results from the two studies could be that when the levels of outdoor pollution has been reduced (2010 year study), air pollution from indoor sources becomes more important and then better ventilation in the classrooms, with lower values of CO_2_, leads to less SBS symptoms. Ventilation is an important factor in the indoor environment, especially in schools. A review of epidemiological studies on ventilation in buildings has concluded that higher ventilation rates in offices were associated with reduced prevalence of SBS symptoms [33]. One recent study has reported that at 1000 ppm CO_2_, compared to a CO_2_-level of 600 ppm, decision-making performance was reduced [34].

In our current study from March 2010, the mean temperature was 18.6 °C indoors and 2.8 °C outdoors. In the previous school study from December 2004 [26], the mean temperature was 14.7 °C indoors and −1.8 °C outdoors. In both surveys, the measurements were performed during heating season but not in the same month, so they were not directly comparable with each other. The higher classroom temperature in 2010 as compared to 2006 could partly be due to improved heating systems.

The indoor and outdoor PM_10_ levels were 129 µg/m^3^ and 168 µg/m^3^ respectively. These levels are higher than the WHO health based standard for PM_10_ of maximum 50 µg/m^3^ as a 24-h mean value [31]. In our study, we found that both indoor and outdoor PM_10_ was associated with increased incidence and decreased remission of most types of SBS symptoms. We found no previous school environment study on SBS symptoms in relation to indoor or outdoor PM_10_ levels, but one study reported an association between PM_10_ levels and reduced nasal patency [35]. Moreover, some studies have reported that ambient particulate matter can cause airway inflammation and oxidative stress [36], [37]. Our findings of both increased incidence and decreased remission of mucosal symptoms associated to PM_10_ are well in accordance with other data from schools [35]–[38]. Since we found health associations for PM_10_ only in the longitudinal analysis, our results indicates a need for more longitudinal school environment studies. Recently there has been a focus on fine particles (PM_2.5_) in ambient air in China. In Taiyuan, the first measurements of PM_2.5_ was performed one year after our study was finished (2013). Data from this year indicate that about 50% of the PM_10_ in outdoor air in Taiyuan consist of PM_2.5_. However, it is likely that the proportion of PM_2.5_ in indoor particles in classrooms in Taiyuan is somewhat lower, since indoor sources in schools mainly generate larger particles (PM_10_) to the classroom air [35], [39].

We found that both indoor and outdoor SO_2_ were positively associated with symptoms improved when away from school in the cross-sectional analysis. In the longitudinal analysis, outdoor SO_2_ was positively associated with new onset of dermal symptoms and negatively associated with remission of any mucosal symptoms. One study from Taiwan, China, found that the prevalence of allergic rhinitis was significantly associated with SO_2_ levels (OR = 1.43) [40]. A previous epidemiological study from four cities in China found that ambient levels of SO_2_ were positively associated with children’s respiratory symptoms [41]. In the previous school environment study from the same schools, we found a positive association between indoor levels of SO_2_ and prevalence of general, mucosal and skin symptoms [26]. In the current study we found a positive association between SO_2_ levels and incidence of skin symptoms. In contrast, in the previous school study from 2004 we found a negative association between SO_2_ levels and incidence of skin symptoms. We have no explanation to this discrepancy, which could be a chance finding.

In our study, we found that outdoor NO_2_ levels were associated with SBS. There were positive associations between NO_2_ concentration and increased incidence of skin, mucosal and general symptoms and a decreased remission of school-related symptoms. However, in the cross-sectional analysis we found a negative association between outdoor NO_2_ and prevalence of skin symptoms, which could be a chance finding. One previous study have demonstrated that an association between NO_2_ levels and prevalence of nasal catarrh, eye symptoms and eczema in 5–14 years old children [42]. In the previous Taiyuan school study from 2004, we found that NO_2_ levels in the classrooms were positively associated with prevalence of mucosal symptoms and symptoms improved if away from school [26].

It is well known that O_3_ can cause mucosal symptoms from the eyes, nose and lower respiratory tract. The associations between NO_2_ and O_3_ is complex, since NO from fresh traffic exhaust reacts with O_3_ to form NO_2_ but with an interaction of other pollutants such as volatile organic compounds (VOC) and particles. In the cross-sectional analysis in our study, indoor O_3_ concentration was positively associated with skin symptoms, only. Other school studies have reported associations between O_3_ and skin symptoms. In one Spanish study, exposure to O_3_ was associated with increased prevalence of skin rashes [43]. In another French study, a moderate increase in long-term exposure to background O_3_ was associated with an increase of atopic indicators in children [44].

In our current study, girls had less mucosal and skin symptoms and lower incidence of skin symptoms. Many previous studies have reported that women report more SBS than men [45], [46]. In the previous school study from the same school, we did not find any associations between gender and SBS. Further studies are needed on gender differences in SBS symptoms in Chinese school children.

In conclusion, SBS symptoms were common in school children in Taiyuan City, China, and symptoms increased during the two-year follow-up period from 2010 to 2012. Parental asthma and allergy (heredity) and pollen or pet allergy (atopy) can be risk factors for both prevalence and incidence of SBS symptoms which could indicate similar mechanisms behind SBS and development of asthma and allergy. Indoor and outdoor levels of air pollutants coming mainly from the outdoor environment, such as PM_10_, SO_2_ and NO_2_ were associated with prevalence, incidence and remission of SBS symptoms in school children. Moreover, we could demonstrate a beneficial effect of increased ventilation flow, measured as lower indoor CO_2_-levels on SBS. Despite the reduced levels of outdoor air pollution in Taiyuan in recent years, impaired health effects of the air pollution can still be detected. This indicates a need for further reduction of pollution levels in Shanxi province. Finally, our study suggests that longitudinal studies, including both incidence and remission data, are needed to evaluate health effects of indoor and outdoor air pollution in schools.
